# Slow art plus: developing and piloting a single session art gallery-based intervention for mental health promotion via a mixed method waitlist randomized control trial

**DOI:** 10.3389/fpubh.2024.1238564

**Published:** 2024-05-13

**Authors:** Andy Hau Yan Ho, Stephanie Hilary Xinyi Ma, Jing Ting Ng, Ping Ying Choo, Geraldine Tan-Ho, Karen Chuan Ling Pooh, Alicia Teng

**Affiliations:** ^1^Action Research for Community Health Laboratory, Psychology Program, School of Social Sciences, Nanyang Technological University, Singapore, Singapore; ^2^Lee Kong Chian School of Medicine, Nanyang Technological University, Singapore, Singapore; ^3^Palliative Care Centre for Excellence in Research and Education, Singapore, Singapore; ^4^Department of Clinical, Educational and Health Psychology, University College London, London, United Kingdom; ^5^Community & Access, National Gallery Singapore, Singapore, Singapore

**Keywords:** social prescribing, gallery and museum collections, mindfulness, participatory art, self-compassion, psychological well-being

## Abstract

**Introduction:**

The current study builds on the expertise of National Gallery Singapore and Nanyang Technological University Singapore (NTU) in developing and piloting an enhanced version of the Slow Art program, namely “Slow Art Plus” for mental health promotion.

**Methods:**

A single-site, open-label, waitlist Randomized Control Trial (RCT) design comprising of a treatment group and waitlist control group was adopted (ClinicalTrials.gov ID: NCT05803226). Participants (*N* = 196) completed three online questionnaires at three timepoints: baseline [T1], immediately post-intervention/s baseline [T2], post-intervention follow-up/immediately post-intervention [T3]. Qualitative focus groups were conducted to evaluate program acceptability.

**Results:**

A mixed model ANOVA was performed to understand intervention effectiveness between the immediate intervention group and waitlist control group. The analyses revealed a significant interaction effect where intervention group participants reported an improvement in spiritual well-being (*p* = 0.001), describing their thoughts and experiences (*p* = 0.02), and nonreacting to inner experiences (*p* = 0.01) immediately after Slow Art Plus as compared to the control group. Additionally, one-way repeated measure ANOVAs were conducted for the intervention group to evaluate maintenance effects of the intervention. The analyses indicated significant improvements in perceived stress (*p* < 0.001), mindfulness (p < 0.001) as well as multiple mindfulness subscales, active engagement with the world (*p* = 0.003), and self-compassion (*p* = 0.02) 1 day after the completion of Slow Art Plus. Results from framework analysis of focus group data revealed a total of two themes (1: Experiences of Slow Art Plus, 2: Insights to Effective Implementation) and six subthemes (1a: Peaceful relaxation, 1b: Self-Compassion, 1c: Widened Perspective, 2a: Valuable Components, 2b: Execution Requisites, 2c: Suggested Enhancements), providing valuable insights to the overall experience and implementation of the intervention.

**Discussion:**

Slow Art Plus represents a unique approach, offering a standardized, multimodal, single-session program that integrates mindfulness and self-compassion practices, as well as reflective and creative expressions with Southeast Asian art. It demonstrates potential in meeting the mental health needs of a wide range of individuals and could be readily incorporated into social prescribing initiatives for diverse populations.

## 1 Introduction

### 1.1 Background

According to the World Health Organization, there is a concerning rise in poor mental health and the prevalence of mental illness worldwide. The prevalence of mental disorders has increased over 13% over the past decade, with depression emerging as one of the prominent cause of disability and suicide as a leading cause of death (1). Similar trends have also been observed in Singapore, as depression and suicide have emerged as two major mental health challenges faced by local society (2). While some factors contributing to poor population mental health could be linked to stress and the inability to cope with life demands (3), the COVID-19 pandemic, with its associated disruptions and isolation, has had a widespread negative impact on mental well-being (4). To address and prevent a looming public mental health crisis, much effort has been placed on mental health promotion in many developed countries in the past decade. Mental health promotion endeavors to minimize the presence of risk factors, enhance protective factors, and promote healthy behaviors which could minimize the risk of developing a diagnosable mental disorder (5). Interventions designed to promote mental health and enhance overall well-being are best implemented within contexts where an individual reside, work, and flourish (6). These interventions encompass a wide range of initiatives, including but not limited to mental health programs implemented in schools and workplaces, social support initiatives, as well as community engagement efforts that uplift the psycho-social determinants of health such as resilience, kindness, and social connectedness.

Mental health promotion in the community through the intersection of mindfulness, self-compassion, and art-based intervention has immense potential to improve individuals’ well-being. Mindfulness, defined as the non-judgmental acceptance of one’s moment-to-moment experience, has been extensively linked to improved overall mental well-being (7). Self-compassion entails actively comforting and soothing oneself with a sense of understanding, recognizing that suffering is a shared human experience (8). According to a meta-analysis by Ferrari et al., self-compassion interventions has resulted in significant improvement in well-being, including mindfulness, stress, anxiety and depression (9). Meditation and mindfulness-based approaches have been practiced across centuries, offering solace and benefits to countless individuals. Although it is not formally established that visual arts engagement is related to mindfulness, there is evidence that point toward shared characteristics between both activities (10, 11). Both activities demand engagement in the present moment, foster a state of flow characterized by intrinsic motivation and absorption, and promote the cultivation of mindfulness in daily life (12). Past research has shown that mindfulness practices enhance mental habits conducive to creativity (13) which may result in richer art-viewing experiences and expressive artwork among children and adults (14). The integration of art appreciation and mindfulness extends beyond the gallery, where such practices were proposed to support meaning-making and the development of practical wisdom among business students, suggesting the role of mindful art appreciation in supporting management education (15, 16).

A growing body of international research has demonstrated the positive impact of arts-based interventions on health promotion and the management of various health conditions (17, 18). Systematic reviews of art-based interventions have indicated that participation in a range of art forms can effectively promote better quality of life, social well-being, and psychological health (19). In addition, the use of arts in mental healthcare settings was beneficial in improving communication skills, stimulating creativity, and supporting behavioral changes (20). Moreover, large population-based studies documented significant associations with engagement in cultural activities and better physical and psychological health (21). In Singapore, a nationwide survey conducted in 2016, known as the Arts for Aging Well Study revealed that active participation in artistic activities and exposure to esthetic experiences had a significant impact on the psychological health, social connections, and spiritual well-being of both adults and older adults (22). This study served as inspiration for a series of local intervention studies. These studies utilized artmaking, storytelling, and creative heritage spaces as therapeutic tools, demonstrating effectiveness in enhancing psychological resilience and quality of life, as well as reducing loneliness among a spectrum of general population groups including community dwelling youths, older adults, informal family caregivers, and professional care workers (23–25). This body of research highlights the importance of further investigation and expansion of art-based interventions utilizing a variety of creative mediums and spaces. This expansion is crucial for extending mental health promotion to larger populations and communities, both locally and internationally. Potential mechanisms underlying the effectiveness of participatory art-based approaches could be explained by the Conceptual Framework for the Roles of the Arts and Humanities for Human Flourishing (26). The model posits that engagement in art encompasses four key dimensions of immersion, embeddedness, socialization, and reflectiveness which supports human flourishing.

Mindfulness, self-compassion, and art-based interventions have independently demonstrated their efficacy in enhancing psychological well-being. However, the confluence of mindfulness and self-compassion within the realm of art-based interventions has yet to be fully explored. In recent years, a global movement coined “Slow Art” has emerged in the field of arts and health promotion. Given its promising potential for fostering meaningful engagement with art, the Slow Art movement, as a gallery-based intervention has gained a significant foothold in numerous museums worldwide (27–32). “Slow Art” aims to foster a deeper esthetic experience for art viewers, thereby enabling them to realize the benefits that the arts have to offer (32). This is especially important given the fact that research has shown that the typical visitor to an art gallery only spends 27 to 33 s observing a single artwork (33). According to Phyl Terry, the founder of Slow Art Day, there exists a lack of knowledge and disconnection among some when it comes to appreciating art. By intentionally slowing down the pace of art appreciation, this deliberate approach allows for a renewed engagement with the artwork, enabling individuals to develop a deeper connection with artistic expressions (34). As such, the slow art experience is characterized by a unique approach to art appreciation which involves an immersive encounter between the viewer and the artwork, thereby encourages prolonged periods of focused attention and contemplation (35, 36). This experience is inherently intertwined with mindfulness, where individuals are required to be fully attentive to the artwork, cultivating an attitude of non-judgmental awareness and openness which has beneficial effects on one’s psychological well-being (37). Although limited, there is a growing body of literature that examines the relationship between “Slow Art” and self-compassion. Engaging in “Slow Art” nurtures social connectedness (38) which is linked to the sense of shared humanity inherent in self-compassion (8). Promoting common humanity is crucial to booster well-being in the wake of the COVID-19 pandemic as many suffered in isolation during lockdowns and social distancing. The incorporation of self-compassion to the experience of “Slow Art,” which has empirically been proven to improve well-being (39, 40), could further enhance social connectedness and potentially create a safe and nurturing environment for participants to explore their thoughts, emotions, and respond to their experiences with kindness. This study is a first-of-its-kind to integrate self-compassion in “Slow Art.”

National Gallery Singapore (Gallery) is committed to audience development and growth. It applies an Audience Engagement Framework in creating and evaluating its arts activities with communities (41). This framework maps out areas of growth for its audience participants across four dimensions as described in Table 1. Recognizing the vast mental health concerns that accompany COVID-19, the Gallery had developed its own Slow Art program in 2020 with the aim to provide interested individuals with a meaningful platform for social connection, conversation, and art appreciation through an online visual art experience. The Slow Art program utilizes artwork from The Care Collection (42), a collaboration between the Gallery and Singapore Art Museum (SAM) to curate a collection of artworks thematically organized for program that supports the well-being of participants. The 60-min single-session Slow Art Online program focuses on one artwork through a series of guided observation exercises followed by facilitated group reflections. Since its launch in April 2020, National Gallery Singapore’s Slow Art series of program has reached close to 950 participants, and results from over 360 feedback surveys show an average satisfaction score of close to 90%. Despite such success, no formal evaluation has been conducted to assess the effectiveness of the Gallery’s Slow Art program for mental health promotion, and this is reflective of the international Slow Art community as there is a scarcity of empirical research to date that examine Slow Art’s efficacy and mechanisms in fostering participants’ well-being.

**Table 1:** Audience engagement framework by National Gallery Singapore.

**Cognitive Dimension 1:** Visual Literacy & Critical Thinking	**Social Dimension 1:** Social Relations	**Personal Dimension 1:** Confidence, Autonomy & Fulfillment	**Cultural Dimension:** Attitude and Commitment to Art
**Cognitive Dimension 2:** Creativity & Innovation	**Social Dimension 2:** Civic Responsibility	**Personal Dimension 2:** Resilience	
**Cognitive Dimension 3:** Knowledge of Art History and Art Theory			

The current study builds on the expertise of Gallery and the Action Research for Community Health Laboratory of Nanyang Technological University Singapore (NTU), in developing and piloting an enhanced version of the Gallery’s Slow Art Online program, namely “Slow Art Plus” for mental health promotion. In practice, the NTU clinical research team has worked closely with the Gallery’s team in reviewing, refining, and co-creating the existing Slow Art program with an infusion of mindfulness and self-compassion practices with reflective and creative expressions that align with The Care Collection and the foundational construct of self-care. Adopting a participatory action research paradigm (43), perspectives and inputs from all relevant stakeholders including the staff, docents, and volunteers of the gallery, as well as NTU mindfulness trainers, and self-compassion experts were elicited to support intervention development. This approach helps to secure support, ownership, active involvement, and the long-term sustainability of the program. The specific objectives of the study are in four-folds:

1) To develop a standardized 90-min single-session Slow Art Plus program that integrates: (a) slow looking; (b) mindfulness meditation; (c) self-compassion activities; (d) reflective-creative expressions; and (e) dyadic sharing, to form a holistic mental health promotion intervention.
2) To assess the effectiveness of Slow Art Plus for reducing participants’ perceived stress (primary outcomes).
3) To assess the effectiveness of Slow Art Plus for enhancing participants’ self-awareness, self-care capacity, psychological resilience, and quality of life (secondary outcomes).
4) To assess the acceptability of the standardized Slow Art Plus protocol for large scale implementation in Singapore and greater Asia.

## 2 Materials and methods

### 2.1 Research design

A single-site, open-label, waitlist Randomized Control Trial (RCT) design comprising two arms: (i) treatment group and (ii) waitlist control group, was adopted to evaluate the efficacy of Slow Art Plus in reducing stress while promoting self-awareness and self-care. A waitlist RCT design is valuable as it allows for a controlled comparison between the intervention group and the wait-list control group, while providing control group participants with access to the intervention promptly. A mixed method approach to data collection including quantitative assessments and an embedded qualitative focus group was utilized for a holistic evaluation of the program and implementation processes. This research protocol was registered on ClinicalTrials.gov (NCT05803226).

### 2.2 Sampling

The proposed sampling frame comprised 200 participants openly recruited from the community and via social media platforms. Inclusion criteria included individuals with the ability communicate in English as the sessions and assessments were implemented in English, as well as to provide informed consent. Exclusion criteria included individuals who were suffering from depression or other major mental health conditions. Mental health conditions were considered an exclusion criterion due to its potential role as a confounding factor for the outcomes of psychosocial well-being. The exclusion was also made to prioritize the well-being of these individuals as there were moments of introspection, reflection, and sharing life stories which may increase the risk of emotional distress. Prospective participants were invited to disclose any formal mental health diagnoses before completing the registration process. Sample size was based on power calculations. With 80% power to detect an effect size of 0.4 (25) based on the primary outcome of stress, as measured by the Perceived Stress Scale (44) in the current study, at 5% level of significance (two-tailed test), a total of 200 participants were required.

### 2.3 Procedures

Participant recruitment was carried out over a 2-month period from September to October 2022 with 12 consecutive and overlapping rounds. Each round involved approximately 20 participants, with 10 randomly assigned to the intervention group and 10 to the waitlist control group. Each round lasted for 3 days. Open recruitment for community-dwelling adults were conducted through event posters strategically placed at the Gallery as well as e-posters disseminated through social media platforms such as Facebook and Instagram. For each recruitment round, prospective participants indicated their interest in the study through an online registration form on Qualtrics. Following this, a designated member of the research team screened prospective participants for eligibility and contacted them regarding the research study and scheduling, and addressed any questions they had about the study.

Consenting participants completed an electronic informed consent form and were invited to complete an electronic baseline assessment prior to Day 1 of the program [T1]. Upon completion of the baseline assessment, successfully recruited participants were randomly allocated into the treatment group (*n* = 10) or waitlist control group (*n* = 10). Participants in the treatment group participated in the 90-min Slow Art Plus program on Day 2 and completed a post-intervention assessment immediately after the program [T2]. One day later, participants in the treatment group completed a follow-up assessment on Day 3 [T3]. Concurrently, participants in the waitlist control group completed a second baseline assessment on Day 2 [T2]. Thereafter, they participated in the same 90-min Slow Art Plus program and completed a post-intervention assessment immediately after the program on Day 3 [T3]. As this was a 90-min, single-session intervention, a three-day timeframe for evaluation was deemed appropriate to assess impact. Moreover, due to the dynamic nature of the outcome variables such as perceived stress (45), a one-day period was deemed suitable for the waiting period.

**Figure 1:**
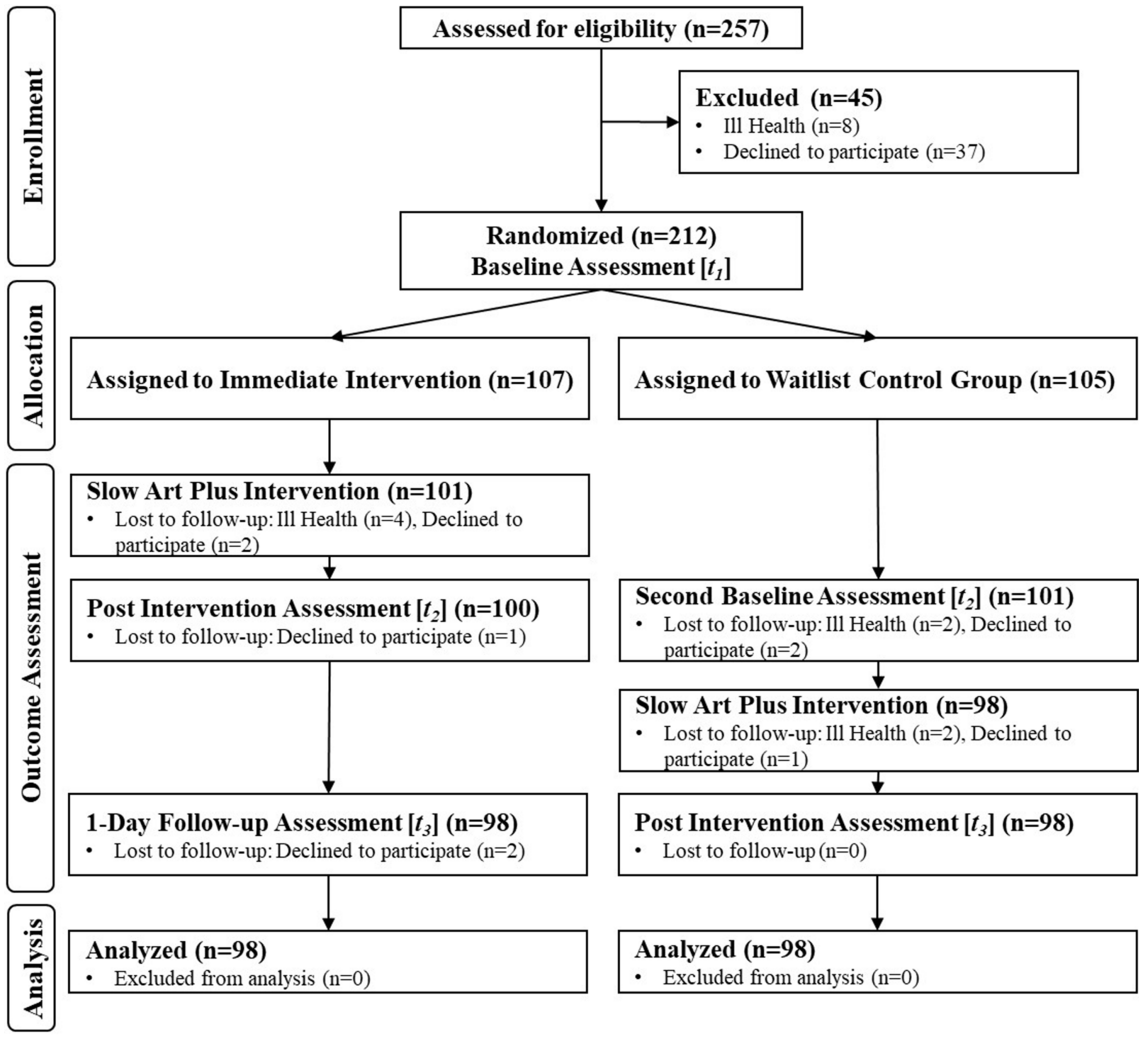
CONSORT study flow.

Additionally, participants in selected rounds were invited to take part in a semi-structured acceptability focus group study immediately after T3 assessments. These groups were chosen based on the availability of activity rooms at the gallery for the focus group interviews, which was a logistical consideration. Each participant received an SGD$20 (Approximately USD$15) monetary incentive upon completing all 3 assessments, and those who were invited to participate in the focus group study received a further SGD$20 incentive. Study procedures are shown in Figure 1.

### 2.4 Randomization

Simple randomization was employed for each recruitment round. This process involved utilizing an allocation sequence derived from a computer-generated list of random numbers ranging from 1 to 20. Upon obtaining informed consent and baseline assessment, participants were randomly assigned a unique number from the sequence. Participants whose numbers corresponded to the first 10 slots in the sequence were allocated to the immediate intervention group, whereas participants whose numbers corresponded to the last 10 slots were allocated to the waitlist control group.

### 2.5 Intervention design

The Slow Art Plus program involved a full 90-min in-person engagement, one that was built upon the original 60-min online Slow Art program with the added layers of mindfulness and self-compassion practices, as well as a curated series of response art activities that foster symbolic dialogs and emotional esthetic experiences between participants and the selected artwork in the physical spaces of the gallery. Each Slow Art Plus session comprised of 6–12 participants and was led by one facilitator.

The first 10-min of the session involved an introduction of the program and its ground rules, as well as a brief psychoeducation on the intersection of slow art, mindfulness, and self-compassion practices. This was followed by a 10-min brief check-in and mood-setting exercise involving all participants to introduce themselves and share one act of self-kindness in the past week. Thereafter, the facilitator led a 10-min mindfulness meditation on affectionate breathing, allowing participants to attain a state of calming openness with greater somatic and emotional clarity for slow-looking. This was followed by a set of 30-min slow looking activity with one selected artwork (title and description concealed), including: (a) crafting a title with a one sentence description that captures the viewers’ emotional response to the artwork with a focus on loving kindness, (b) creating a soundscape or a music playlist that resonates with the viewers’ response and the imagined stories behind the artwork, and (c) sketching a response art to the selected artwork which allows perspective widening by facilitating dialog between the viewer, the art, and the artist. Thereafter, a 10-min dyadic sharing took place with participants, followed by a 4-min group conversation where each dyad had 1 min to share their joint collective experience to the bigger group, moderated by the facilitator. Artwork reveal and sharing of its self-care implications were provided and lasted 6 min. The final segment of the session involved a check-out and closure activity with a brief supportive touch meditation which lasted for 10 min. A breakdown of the Slow Art Plus Protocol is provided in Table 2.

**Table 2:** Slow art plus protocol.

Activity	Time
1 Introduction and Psychoeducation on Slow Art, Mindfulness & Self-Compassion	10 min
2 Participant Check-in & Sharing of 1 act of Self-Kindness	10 min
3 Mindfulness Meditation on Affectionate Breathing^a^	10 min
4 Slow Looking at 1 selected Artwork with 3 standardized activities: a) Crafting an Artwork Title with viewers’ emotional response description b) Developing a Soundscape or Music Playlist that resonates with viewers’ emotional response. c) Sketching a Response Art to facilitate symbolic dialog & emotional esthetic	30 min
5 Dyadic Sharing and Group Conversation	14 min
6 Artwork Reveal and Sharing of its Self-Care implication	6 min
7 Check-out and Closure with a Supportive Touch Meditation	10 min
Total	90 min

^a^Affectionate breathing is a technique that regulates emotions by focusing on the breath and fostering a compassionate connection with oneself.

Specifically, the inclusion of both passive and active art engagement activities was informed by past literature; where the appreciation of artworks elicited an emotional and intellectual exchange (46), whilst the active engagement of sketching an artwork encouraged creative control (47). The addition of the music playlist creation activity provided an additional layer of cognitive stimulation, evoking memories and emotions (48). Furthermore, the amalgamation of mindfulness and self-compassion activities deepened the engagement with the artwork as well as one’s emotionality and awareness in the immediacy of the esthetic experience. Altogether, the scaffolding of these multi-modal intervention elements provided participants with an immersive and holistic experience for self-discovery and self-care.

#### 2.5.1 Choice of artwork

**Figure 2:**
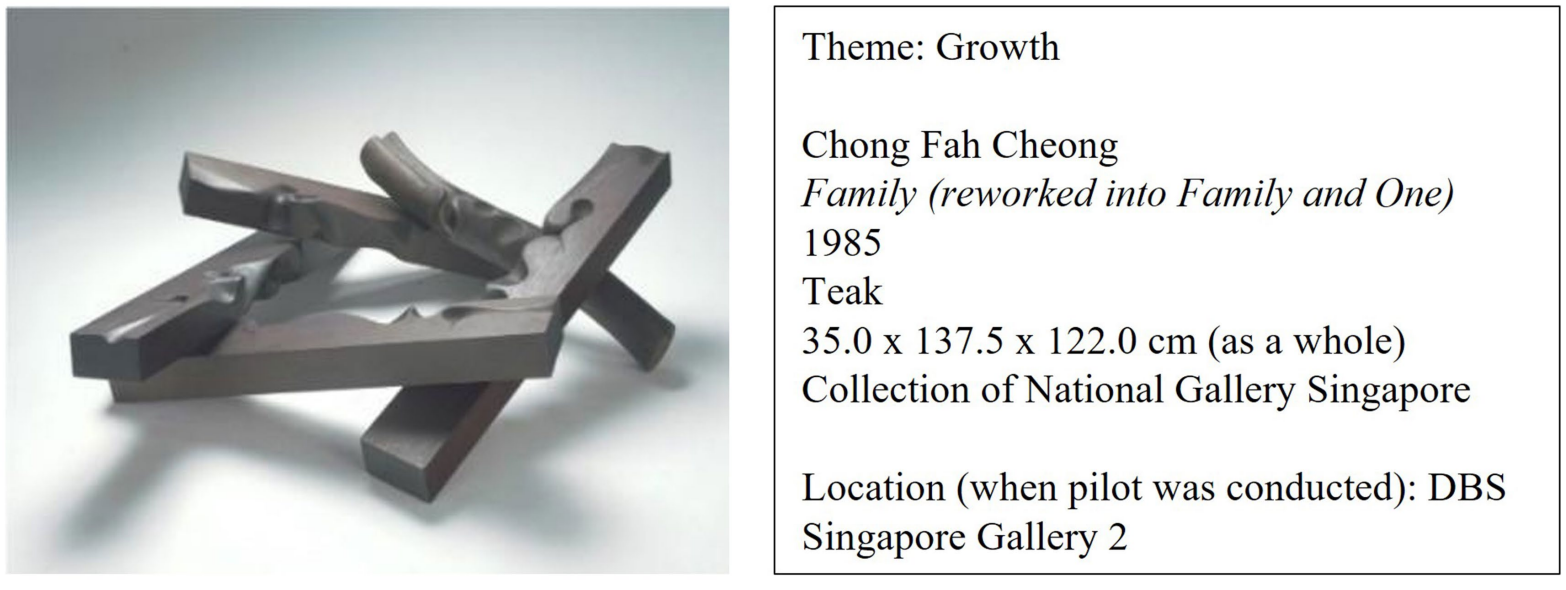
Sculpture chosen for the slow art plus intervention.

*Family (reworked into Family and One)* is a wooden sculpture created by Singapore’s pioneer sculptor Chong Fah Cheong. The sculpture is made of five carved wooden planks which lean against each other to form an interlocked coil. Initially titled *Family*, Chong reworked this sculpture into *Family and One* by adding a fifth plank with rounded edges and a distinct organic form. This additional plank stood out from the original four, expanding on the notion of family. *Family (reworked into Family and One)* presents the viewer with a visual idiom of support, teamwork, and togetherness. Its title links directly to the theme of ‘family’ and all its associated experiences. By reflecting on the sculpture, the viewer is invited to think about supportive loved ones, along with how one can also be a supportive loved one to others. Refer to Figure 2 for more information.

**Figure 3:**
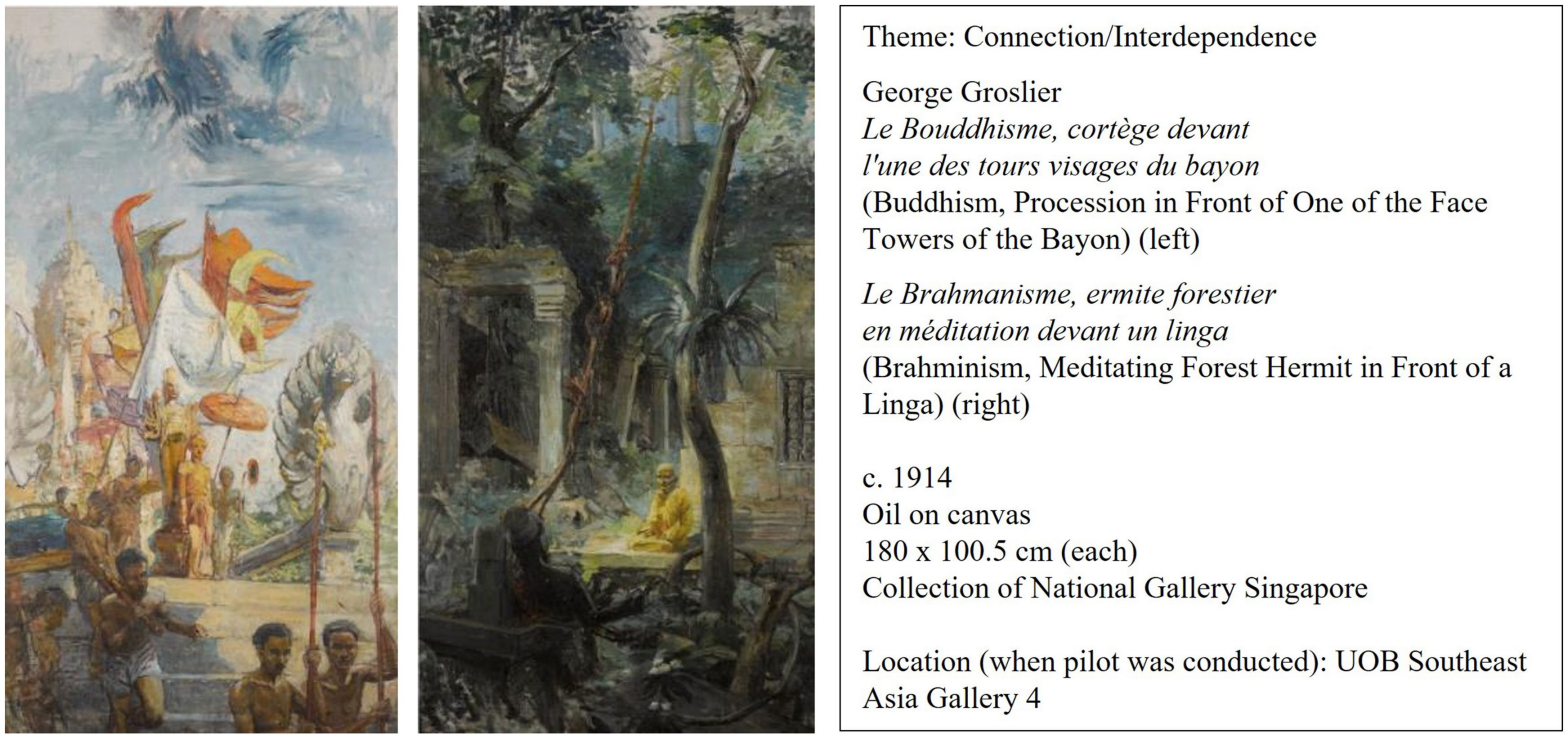
Paintings chosen for the slow art plus intervention.

*Buddhism, Procession in Front of One of the Face Towers of the Bayon* and *Brahminism, Meditating Forest Hermit in Front of a Linga* are a pair of oil paintings made by George Groslier. This pair of paintings was likely intended by Groslier to be a commissioned work for the Throne Hall of the Royal Palace of Cambodia with the subjects being Buddhism, Brahmanism, and the relation between the two. The first painting (Buddhism) presents the viewer with the theme of community and celebration. The figures in the painting are participating in a religious ceremony which pays homage to their respective gods. Colorful flags and ornate grand statues add to the grandiosity of this festive experience, under the sunny and clear sky. In contrast, the second painting (Brahminism) presents the viewer with a somber, dark atmosphere of solitude. A single, solitary hermit meditates amidst the ruins of a temple, surrounded by the quiet of the forest. Spirituality, nature, and aging are reflective themes that can be derived from observing this painting. As a pair, the paintings present the viewer with both the celebratory and solitary aspects of life. The social and the personal come to the fore as one contemplates the meaning of life with all its myriad forms of experience. They also present the viewer with different ways of life, as led by others from different cultures, in different parts of the world. Refer to Figure 3 for more information.

### 2.6 Outcome measures

Primary outcomes include participants’ reported levels of perceived stress, of which was assessed by a modified version of the Perceived Stress Scale (PSS) (44). The PSS comprises 10-items rated on a 5-point Likert scale and clustered into 2 subscales of perceived helplessness (e.g., “In the past couple of days, how often have you felt nervous and stressed today”), and perceived self-efficacy (e.g., In the past couple of days, how often have you felt that things were going your way”). The PSS possesses strong internal validity, reliability, and cross-cultural applicability.

Secondary outcomes include participants’ self-awareness, self-compassion, psychological resilience, and quality of life. First, self-awareness was assessed by the Five Facet Mindfulness Questionnaire Short Form (SF-FFMQ) comprising 20 items clustered into 5 subscales: (i) observe, (ii) describe, (iii) act with awareness, (iv) non-judging of inner experience, and (v) non-reactivity to inner experience (49). Second, self-care capacity is assessed by the Self-Compassion Scale - Short Form (SCS-SF) comprising 12 items clustered into 3 subscales: (i) self-kindness- self-judgment, (ii) common humanity- isolation, (iii) mindfulness- over-identification (50). Third, psychological resilience was assessed by the Ego-Resilience Scale-11 (ER-11) comprising 11-items clustered into 3 subscales: (i) active engagement with the world, (ii) repertoire of problem-solving strategies, and (iii) integrated performance under stress (51). Fourth, quality of life was measured by the Single-Item Quality of Life Scale (SI-QOLS) (52). Finally, spiritual well-being was assessed by the 12-item functional assessment of chronic illness therapy-spiritual well-being scale (FACIT-SP-12) which were clustered into 3 subscales of meaning, peace, and faith (53). All secondary outcome measures including the SF-FFMQ, SCS-SF, ER-11, SI-QOLS, and FACIT-SP-12 possessed strong internal validity, reliability, and cross-cultural applicability. Demographic information including age, sex, ethnicity, and socio-economic status were collected from participants at baseline.

To assess acceptability of the Slow Art Plus program, three acceptability focus groups were conducted with the aim to elicit: (a) experiences of the intervention, (b) feedback on intervention components, (c) information on intervention effectiveness, (d) facilitators and barriers to sustained engagement in the program, (e) factors to promote future participation to a wider audience. A semi-structured interview guide was developed with questions including, “What was your experience like with the Slow Art Plus program?,” “How has the program components (e.g., slow looking, mindfulness meditation, self-compassion activities, reflective-creative expressions, dyadic sharing, gallery space, facilitation) influenced your overall experience?,” and “What did you find most/least helpful about Slow Art Plus?.” All focus groups were conducted by a member of the research team and was recorded and transcribed verbatim for analyses. In addition, participant’s written responses of their experience of the slow art plus study as well as satisfaction scores were collected at the end of the post-session assessment with the quantitative measures. An example of the prompts includes “What did you appreciate about this program?,” “What could be improved about this program,” “Have you observed any changes in yourself during or after the program? Please elaborate on your experience,” and “Do you have any thoughts, comments, or feedback that you would like to share?”

### 2.7 Data analysis

The quantitative data were managed and analyzed using the IBM Statistical Package for the Social Sciences (SPSS) v25 statistical analysis software (Armonk, NY, United States). The immediate intervention and waitlist control groups were compared on the primary outcomes of perceived stress and secondary outcomes of mindfulness, self-compassion, psychological resilience, spiritual well-being, and quality of life. Firstly, a mixed model analysis of variance (ANOVA) was conducted to analyze intervention effects between the immediate intervention group and control group at T1 and T2 for each outcome variable. For measures with statistically significant interactions, follow-up tests for simple main effects using a one-way ANOVA for group effects or a repeated measure ANOVA for time effects were conducted. Only significant simple main effects were reported in the manuscript for brevity. Secondly, maintenance effects of the intervention were performed using repeated measure ANOVA with the immediate intervention group at T2 and T3 with baseline assessment (T1). *Post-hoc* pairwise comparisons were conducted for significant findings with Bonferroni corrections to reduce the likelihood of false positives. Assumptions testing was conducted to check for any violations of assumptions for outliers, normal distributions, homogeneity of variance and sphericity. There was a violation of normality for stress, quality of life, self-compassion, and spiritual well-being. As ANOVA tests are robust against normality assumption violation (54), the reporting of the results was kept consistent using parametric tests. Mann–Whitney U test and Friedman test with follow-up Wilcoxon Signed-Rank Test were conducted for variables that violate the normality assumption and can be found in the supplementary resources. To assess multicollinearity among the independent outcome variables, a Pearson correlation analyses was conducted. The results indicated no evidence of multicollinearity between the variables (*r* < 0.9). A high correlation was observed for the perceived stress scale and one of its subscales of perceived helplessness (*r* = 0.95–0.96). While a MANOVA could have been an alternative approach to manage multiple outcome variables, ANOVAs were employed instead of MANOVAs due to the exploratory nature of the study which sought to investigate the intervention’s effects on individual outcome variables rather than their interrelationships (55). Nonetheless, a supplementary mixed MANOVA and repeated MANOVA analysis were conducted and the findings from these analyses were consistent with the results obtained from the ANOVAs, providing further support for the current analysis. In case of any violation of the test of sphericity while conducting the tests, the Greenhouse–Geisser correction was used. Baseline demographic information, recruitment, and dropout rates, as well as the appropriate means, F ratio, *p* value and effect size estimates for the ANOVA analyses were reported.

The qualitative data were analyzed using framework analysis which involved both inductive and deductive approaches to systematically examine the data for conceptual themes and theme categories that illuminate program acceptability. Framework analysis is a valuable tool in health policy research as it addresses specific research questions with pre-defined issues to explore, contributing to evidence-based decision making (56). The analysis involved several steps of (a) data familiarization and line-by-line coding to delineate the central concepts that emerged from the interviews; (b) the formation of themes and sub-themes by combining codes that were conceptually similar; and (c) axial coding to develop and refine possible theme-categories. The preliminary themes were reviewed by the NTU research team, and subsequently presented to the larger research team during regular meetings for further discussion and revision. This process ensured that the qualitative findings were reliable and that there was agreement among researchers in their interpretation of the data. In the final stage, all major categories, themes, and sub-themes were defined and operationalized with supporting quotes from interview transcripts. This led to the development of an overarching framework that highlighted the well-being impact and lessons gleaned from the implementation processes of the Slow Art Plus program. Research rigor and trustworthiness were ensured by adopting strategies such as prolonged engagement with the data, peer debriefing, maintenance of an audit trail, thick descriptions of data, negative case analysis, as well as triangulation of data, investigator, and theory.

## 3 Results

### 3.1 Participant demographics

A total of 225 participants were successfully recruited with 196 participants completing the study. Participants’ age ranged between 18 and 85 years (*M* = 45.9, *SD* = 16.6), with 71% being women participants and predominantly of Chinese ethnicity (88%). Most participants did not have a chronic illness (87%) and nor were receiving any counseling services (92%). There were no significant differences for all demographic measures between the immediate intervention and waitlist control group. Please refer to Table 3 for the full participants’ demographics.

**Table 3:** Participant demographic information.

Demographic characteristic	Immediate Intervention	Waitlist Control
	(*n* = 98)	(*n* = 98)
	Mean (*SD*) or *N* (%) or range	
Age in years, Mean (SD)	47.67 (15.70)	44.06 (17.33)
Age range	21 to 76 years	18 to 85 years
Female	70 (71.4)	69 (70.4)
Male	28 (28.6)	29 (29.6)
Artwork Viewed		
Artwork 01 – *Buddhism, Procession in Front of One of the Face Towers of the Bayon*, and *Brahminism, Meditating Forest Hermit in Front of a Linga*	53 (54.1)	51 (52.0)
Artwork 02 – *Family (reworked into Family and One)*	45 (45.9)	47 (48.0)
Marital Status		
Single/Divorced/Widowed	48 (49)	64 (65.3)
Married	50 (51)	34 (34.7)
Education		
PSLE, GCE ‘N/O/A’, Nitec or Higher Nitec	7 (7.1)	18 (18.4)
Polytechnic Diploma	12 (12.2)	10 (10.2)
Professional Certificate	4 (4.1)	2 (2.0)
Bachelor’s Degree	49 (50.0)	43 (43.9)
Postgraduate Degree	26 (26.5)	25 (25.5)
Ethnicity		
Chinese	86 (87.8)	87 (88.8)
Malay	2 (2.0)	1 (1.0)
Indian	2 (2.0)	6 (6.0)
Others (Austronesian, Bulgarian, Caucasian, European, Filipino, Indonesian, Sinhalese, and Vietnamese)	8 (8.2)	4 (4.1)
Employment Status		
Full-time Employed	36 (36.7)	34 (34.7)
Part-time Employed/ Self-Employed	16 (16.3)	15 (15.3)
Unemployed/Retired/ Student/ Other	46 (46.9)	49 (49.9)
Presence of Chronic Illness		
No	84 (85.7)	86 (87.8)
Yes	14 (14.3)	12 (12.2)
Receiving Counseling Services		
No	89 (90.8)	92 (93.9)
Yes	9 (9.2)	6 (6.1)
		

### 3.2 Quantitative analysis

#### 3.2.1 Between-group analysis: mixed model ANOVA

For the primary outcome of perceived stress, although there was a decrease in perceived stress scores, there were no significant interaction effects between group and time. Similarly for the secondary outcomes of mindfulness, resilience, quality of life, and self-compassion, there were no significant interaction effects. However, there was a significant group and time interaction for spiritual well-being, *F*(1, 194) = 10.99, *p* = 0.001, ⴄ_p_^2^ = 0.054. Follow-up simple main effect for group analysis revealed that there were no differences between the intervention group and waitlist control group at T1 (baseline), but a marginally significant difference at T2 (immediate intervention/s baseline assessment), *F*(1,194) = 3.66, *p* = 0.06, ⴄ_p_^2^ = 0.019. Additionally, simple main effect for time analysis indicated that there were differences in the intervention group (*F*(1, 97) = 25.04, *p* < 0.001, ⴄ_p_^2^ = 0.205), but not the waitlist control group.

Significant interaction effects were also observed in the peace (F(1, 194) = 7.08, *p* = 0.008, ⴄ_p_^2^ = 0.035) and faith (F(1, 194) = 7.07, *p* = 0.009, ⴄ_p_^2^ = 0.035) subscales of the spiritual well-being scale. Follow-up simple main effect for group analysis revealed that there were no differences at T1 between the intervention group and control group for both subscales. However, there was a significant difference between the groups at T2 for the peace (F(1,194) = 6.11, *p* = 0.014, ⴄ_p_^2^ = 0.031) but not the faith subscale. Significant simple main effect of time was reflected in the peace (*F*(1, 97) = 12.77, *p* = 0.001, ⴄ_p_^2^ = 0.116) and faith (*F*(1, 97) = 24.72, *p* < 0.001, ⴄ_p_^2^ = 0.203) subscales for the intervention group, but not the waitlist control group.

Although there were no significant interaction effects on the overall mindfulness scale, there was a significant interaction effect observed in the describing (*F*(1, 194) = 5.96, *p* = 0.016, ⴄ_p_^2^ = 0.030) and nonreacting to inner experience (F(1, 194) = 6.45, *p* = 0.012, ⴄ_p_^2^ = 0.032) subscale. Follow-up simple main effect for group analysis revealed that there were no differences at T1 and T2 between the intervention group and control group for both subscales. Simple main effect of time analysis revealed a significant difference in the intervention group for the describing (*F*(1, 97) = 4.95, *p* = 0.028, ⴄ_p_^2^ = 0.049) and nonreacting to inner experience (F(1, 97) = 7.59, *p* = 0.007, ⴄ_p_^2^ = 0.073) subscale, but not the waitlist control group. Details of the mixed model ANOVA can be found in Table 4. A further exploration of the intervention’s effects over time in the intervention group is reported in the next section.

**Table 4:** Between-group analysis using mixed model ANOVA.

Variables	Immediate intervention (*N* = 98)		Waitlist control (*N* = 98)		Group effect^a^		Time effect^a^		Group x time interaction^a^	
	T1	T2	T1	T2	*f* ratio	*ⴄ_p_^2^*	*f* ratio	*ⴄ_p_^2^*	*f* ratio	*ⴄ_p_^2^*
	Means (*SD)*	Means (*SD)*	Means (*SD)*	Means (*SD)*						
Primary Outcome										
Perceived Stress (PSS-10)	15.10 (6.35)	14.64 (6.75)	15.06 (6.70)	14.13 (7.25)	0.094	<0.001	3.807	0.019	0.436	0.002
Perceived Helplessness	9.58 (4.19)	8.96 (4.41)	9.41 (4.60)	8.64 (4.95)	0.170	0.001	7.013*	0.035	0.074	<0.001
Lack Of Self-Efficacy	5.52 (2.68)	5.68 (2.89)	5.65 (2.85)	5.49 (2.82)	0.007	<0.001	<0.001	<0.001	1.015	0.005
Secondary Outcome										
Mindfulness (SF-FFMQ)	67.35 (8.50)	67.45 (8.68)	67.51 (8.93)	66.56 (8.91)	0.095	<0.001	0.946	0.005	1.457	0.007
Observing	14.91 (2.92)	14.99 (2.89)	14.63 (2.65)	14.69 (2.83)	0.578	0.003	0.239	0.001	0.005	<0.001
Describing	13.56 (2.81)	14.05 (3.15)	13.63 (3.16)	13.37 (2.91)	0.582	0.003	0.527	0.003	5.959*	0.030
Acting With Awareness	13.49 (2.84)	12.86 (2.81)	13.58 (3.11)	13.32 (3.26)	0.469	0.002	8.791*	0.043	1.147	0.008
Nonjudging To Inner Experience	12.52 (3.00)	12.14 (3.25)	12.55 (3.52)	12.30 (3.48)	0.045	<0.001	2.714	0.014	0.102	0.001
Nonreacting To Inner Experience	12.87 (2.31)	13.41 (2.34)	13.11 (2.56)	12.89 (2.47)	0.196	0.001	1.102	0.006	6.448*	0.032
Psychological Resilience (ER11)	3.05 (0.43)	3.06 (0.41)	2.98 (0.50)	2.96 (0.48)	1.903	0.010	0.217	0.001	0.556	0.003
Integrated Performance Under Stress	3.02 (0.63)	2.97 (0.59)	2.95 (0.64)	2.92 (0.57)	0.586	0.003	1.084	0.006	0.089	<0.001
Active Engagement with The World	3.15 (0.56)	3.17 (0.52)	3.01 (0.59)	3.00 (0.58)	4.321*	0.022	0.007	<0.001	1.023	0.005
Repertoire Of Cognitive, Social and Personal Problem-Solving Strategies	2.96 (0.56)	2.97 (0.56)	2.97 (0.59)	2.95 (0.55)	0.005	<0.001	0.034	<0.001	0.306	0.002
Quality of Life (SI-QOLS)	5.22 (1.19)	5.35 (1.18)	5.17 (1.21)	5.15 (1.15)	0.615	0.003	0.632	0.003	1.240	0.006
Self-Compassion (SCS-SF)	3.27 (0.55)	3.30 (0.53)	3.37 (0.68)	3.33 (0.69)	0.534	0.003	0.009	<0.001	1.986	0.010
Self-Kindness, Self-Judgment	3.33 (0.65)	3.33 (0.59)	3.41 (0.79)	3.37 (0.80)	0.388	0.002	0.219	0.001	0.285	0.001
Common Humanity, Isolation	3.24 (0.62)	3.29 (0.65)	3.31 (0.72)	3.30 (0.74)	0.241	0.001	0.223	0.001	0.582	0.003
Mindfulness, Over-Identification	3.24 (0.70)	3.30 (0.67)	3.38 (0.80)	3.31 (0.77)	0.677	0.003	0.027	<0.001	2.781	0.014
Spiritual Well-being (FACIT-SP-12)	32.22 (9.10)	35.00 (7.81)	32.15 (9.77)	32.65 (9.29)	0.948	0.005	22.77**	0.105	10.99**	0.054
Meaning	11.88 (3.40)	12.44 (2.82)	11.80 (3.15)	11.88 (3.01)	0.597	0.003	4.467*	0.023	2.486	0.013
Peace	10.56 (3.56)	11.72 (3.04)	10.47 (3.51)	10.59 (3.37)	1.928	0.010	10.81**	0.053	7.083*	0.035
Faith	9.79 (4.63)	10.84 (4.37)	9.89 (4.68)	10.18 (4.58)	0.187	0.001	22.48**	0.104	7.065*	0.035

*p < 0.05, **p < 0.001; ^a^df: 1,194.

#### 3.2.2 Within-group analysis: repeated measures ANOVA

For the primary outcome, participants reported a significant reduction in perceived stress (*F*(1.86, 180.19) = 14.52, *p* < 0.001, ⴄ_p_^2^ = 0.130) over time. Post-hoc analyses indicated a significant decrease in stress one day after completing Slow Art Plus (T3) (MD = −2.55, 95% CI [−3.92, −1.19], p < 0.001). This finding was also mirrored in the perceived stress subscales where participants reported a reduction in perceived helplessness (*F*(2, 194) = 14.81, p < 0.001, ⴄ_p_^2^ = 0.132; MD = −1.86, 95% CI [−2.75, −0.96], *p* < 0.001) and lack of self-efficacy (*F*(1.85, 178.99) = 7.40, *p* = 0.001, ⴄ_p_^2^ = 0.071; MD = −0.69, 95% CI [−1.34, −0.05], *p* = 0.031) one day after the intervention (T3).

For the secondary outcome, there was a statistically significant change over time indicated in the mindfulness (*F*(2, 194) = 12.56, *p* < 0.001, ⴄ_p_^2^ = 0.115) as well as the observing (F(2, 194) = 12.28, *p* < 0.001, ⴄ_p_^2^ = 0.112), describing (*F*(1.81,176.26) = 7.15, *p* = 0.001, ⴄ_p_^2^ = 0.069), acting with awareness (*F*(1.89, 182.88) = 6.06, *p* = 0.003, ⴄ_p_^2^ = 0.059) and nonreacting to inner experience (F(2, 194) = 7.57, *p* = 0.001, ⴄ_p_^2^ = 0.072) subscales. There was also a significant change over time observed for the active engagement with the world (ER11 subscale; F(2, 194) = 5.85, *p* = 0.003, ⴄ_p_^2^ = 0.057) and self-compassion (F(2, 194) = 4.32, *p* = 0.015, ⴄ_p_^2^ = 0.043). Lastly, there were statistically significant differences in the spiritual well-being (*F*(1.89, 182.84) = 15.22, *p* < 0.001, ⴄ_p_^2^ = 0.136) as well as the meaning (F(2, 194) = 4.33, *p* = 0.014, ⴄ_p_^2^ = 0.043), peace (*F*(1.83, 177.79) = 7.99, *p* = 0.001, ⴄ_p_^2^ = 0.076), and faith (*F*(1.69, 164.06) = 15.77, *p* < 0.001, ⴄ_p_^2^ = 0.140) subscales.

Post-hoc pairwise comparisons revealed that immediately after the session (T2), there was an increase in spiritual peace (FACIT-SP-12 subscale; MD = 1.16, 95% CI [0.37, 1.96], *p* = 0.002). A reduction in acting with awareness (SF-FFMQ subscale; MD = −0.63, 95% CI [−1.21, −0.05], *p* = 0.028) was also observed immediately after the session but returned to baseline levels the next day (MD = 0.65, 95% CI [−0.16, 1.14], *p* = 0.005). Furthermore, there was a significant improvement in nonreacting to inner experience (SF-FFMQ subscale; MD = 0.54, 95% CI [0.063, 1.02], *p* = 0.021) and spiritual well-being (MD = 2.78, 95% CI [1.42, 4.13], *p* < 0.001) including the meaning (MD = 0.56, 95% CI [0.01, 1.12], *p* = 0.047) and faith subscales immediately after the session (T2) and the effect was maintained 1 day after Slow Art Plus (T3). Lastly, 1 day after the intervention (T3), participants reported further improvements in overall mindfulness (MD = 2.84, 95% CI [1.20, 4.47], *p* < 0.001) including the observing (MD = 0.90, 95% CI [0.39, 1.41], *p* < 0.001) and describing (MD = 0.74, 95% CI [0.24, 1.23], *p* = 0.001) subscales, active engagement with the world (ER11 subscale; MD = 0.11, 95% CI [0.02, 0.20], *p* = 0.009), and overall self-compassion (MD = 0.11, 95% CI [0.01, 0.21], *p* = 0.030). Detailed findings from the repeated measure ANOVAs can be found in Table 5.

**Table 5:** One-way repeated measures ANOVA for the immediate intervention group (*n* = 98).

Variables	T1	T2	T3	ANOVA		T1 *vs* T2		T1 *vs* T3	
	Means (*SD)*	Means (*SD)*	Means (*SD)*	*f* ratio	*ⴄ_p_^2^*	95% CI	Mean Differences (T2 – T1)	95% CI	Mean Differences (T3 – T1)
Primary Outcome									
Perceived Stress (PSS-10)	15.10 (6.35)	14.64 (6.75)	12.55 (6.45)	14.52^a^**	0.13	(−0.78, 1.70)	−0.46	(1.19, 3.92)	−2.55**
Perceived Helplessness	9.58 (4.19)	8.96 (4.41)	7.72 (4.47)	14.81**	0.13	(−0.26, 1.50)	−0.62	(0.96, 2.75)	−1.86**
Lack Of Self-Efficacy	5.52 (2.68)	5.68 (2.89)	4.83 (2.48)	7.40^a^*	0.07	(−0.74, 0.41)	0.16	(0.05, 1.34)	−0.69*
Secondary Outcome									
Mindfulness (SF-FFMQ)	67.35 (8.50)	67.45 (8.68)	70.18 (9.00)	12.56**	0.12	(−1.76, 1.55)	0.10	(−4.47, −1.20)	2.84**
Observing	14.91 (2.92)	14.99 (2.89)	15.81 (2.91)	12.28**	0.11	(−0.59, 0.42)	0.08	(−1.41, −0.39)	0.90**
Describing	13.56 (2.81)	14.05 (3.15)	14.30 (3.04)	7.15^a^*	0.07	(−1.03, 0.05)	0.49	(−1.23, −0.24)	0.74*
Acting With Awareness	13.49 (2.84)	12.86 (2.81)	13.51 (3.10)	6.06^a^*	0.06	(0.05, 1.21)	−0.63*	(−0.50, 0.46)	0.02
Nonjudging To Inner Experience	12.52 (3.00)	12.14 (3.25)	12.99 (3.27)	4.23 ^a^*	0.04	(−0.37, 1.12)	−0.38	(−1.23, 0.29)	0.47
Nonreacting To Inner Experience	12.87 (2.31)	13.41 (2.34)	13.58 (2.32)	7.57**	0.07	(−1.02, −0.06)	0.54*	(−1.21, −0.22)	0.71*
Psychological Resilience (ER11)	3.05 (0.43)	3.06 (0.41)	3.11 (0.41)	2.15	0.02	(−0.07, 0.06)	0.006	(−0.13, 0.02)	0.06
Integrated Performance Under Stress	3.02 (0.63)	2.97 (0.59)	3.00 (0.53)	0.40^a^	0.004	(−0.07, 0.16)	−0.46	(−0.12, 0.162)	−0.02
Active Engagement with The World	3.15 (0.56)	3.17 (0.52)	3.26 (0.52)	5.85*	0.057	(−0.10, 0.06)	0.02	(−0.20, −0.02)	0.11*
Repertoire Of Cognitive, Social and Personal Problem-Solving Strategies	2.96 (0.56)	2.97 (0.56)	2.98 (0.56)	0.18	0.002	(−0.11, 0.09)	0.01	(−0.12, 0.07)	0.02
Quality of Life (SI-QOLS)	5.22 (1.19)	5.35 (1.18)	5.32 (1.15)	1.06^a^	0.01	(−0.35, 0.11)	0.12	(−0.33, 0.15)	0.09
Self-Compassion (SCS-SF)	3.27 (0.55)	3.30 (0.53)	3.38 (0.55)	4.32*	0.04	(−0.12, 0.05)	0.04	(−0.21, −0.01)	0.11*
Self-Kindness, Self-Judgment	3.33 (0.65)	3.33 (0.59)	3.43 (0.65)	2.26	0.02	(−0.12, 0.11)	0.003	(−0.24, 0.04)	0.10
Common Humanity, Isolation	3.24 (0.62)	3.29 (0.65)	3.35 (0.64)	2.07	0.02	(−0.16, 0.07)	0.04	(−0.24, 0.03)	0.11
Mindfulness, Over-Identification	3.24 (0.70)	3.30 (0.67)	3.36 (0.67)	2.56	0.03	(−0.19, 0.07)	0.06	(−0.26, 0.02)	0.12
Spiritual Well-being (FACIT-SP-12)	32.22 (9.10)	35.00 (7.81)	33.94 (8.63)	15.22^a^**	0.14	(−4.13, −1.42)	2.78**	(−2.97, −0.46)	1.71*
Meaning	11.88 (3.40)	12.44 (2.82)	12.38 (3.11)	4.33*	0.04	(−1.12, −0.01)	0.56*	(−1.00, −0.00)	0.50*
Peace	10.56 (3.56)	11.72 (3.04)	11.09 (3.15)	7.99^a^**	0.08	(−1.96, −0.37)	1.16*	(−1.25, 0.19)	0.53
Faith	9.79 (4.63)	10.84 (4.37)	10.47 (4.39)	15.77^a^**	0.14	(−1.57, −0.54)	1.05**	(−1.19, −0.18)	0.68*

*p < 0.05, **p < 0.001; ^a^Greenhouse–Geisser correction was used when the data violated the assumption of sphericity.

### 3.3 Qualitative findings

Qualitative responses were collected from participants via three acceptability focus group discussions and qualitative written feedback at the end of the survey. Both data sources were analyzed using a framework analysis and a total of two themes and six subthemes were identified.

#### 3.3.1 Theme 1: Experiences of slow art plus (*n* = 176; mentioned by 176 participants)

Participants in the study reported experiencing various positive impacts of the intervention including a sense of peace and calmness, enhanced self-compassion, and a broader perspective of their life experience.

Subtheme 1a: Peaceful Relaxation (*n* = 85). Participants of the study reported feeling calmer and less stressed after the session which contributed to their overall sense of well-being. For instance, one shared that ‘*I definitely felt more at peace, and more mindful after the program. I could stop thinking about the different stressors I had been facing for just the 90 min, which was very helpful (SAP221, 25-year-old, male)*’. Another participant shared similar sentiments, where *‘I feel calmer and less stressed and more at peace with myself. There seems to be a layer of peace draped over me. I am more aware and cognizant of the need to also take care of myself, and to set aside time for my own self-reflection and exploration* (*SAP087, 22-year-old, female*)’. Some participants found the intervention to be therapeutic. For instance, one participant reported a reduction in bodily tension, where ‘*the stiffness in my shoulders reduced significantly. I also noticed my mind quietening (SAP275 35-year-old, female)’.*

Subtheme 1b: Self-compassion (*n* = 106). Participants demonstrated aspects of self-compassion, including mindfulness, self-kindness, and common humanity. The meditation exercises in the sessions helped participants discover insights that they could incorporate into their daily lives, as this participant mentioned, ‘*when you get connected to your body, you are more confident in yourself because you are aware of what’s happening, it creates a more positive mindset about yourself and what is happening around you, the challenges that you have. That [mindset] helps you to overcome it positively (SAP165, 59-year-old, female)*’. Furthermore, participating in the session with others enhanced their sense of connection, as succinctly expressed by one participant, *‘we get to share our thoughts with each other. It makes me feel like I’m not alone in my struggles and that we probably see the same things in life (SAP083, 30-year-old, female)’*. Participants became more aware of the importance of self-care and made more deliberate effort to prioritize it after the sessions, ‘*I believe it increased my level of self-care. Being a mother, I will splurge money on my children, but I tend not to splurge on myself so after this session, I love flowers so I’m going to the florist and buy myself a bouquet of flowers (SAP187, 61-year-old, female)’.*

Subtheme 1c: Widened Perspective (*n* = 60). Engaging in Slow Art Plus broadened the perspectives of participants. The self-reflective aspects of the intervention helped participants discover meaning in their life experiences, as described by one participant, ‘*there were many self-reflective components where I could look inwards and connect whatever I was experiencing with my inner self (SAP087, 22-year-old, female)*’. Observing the artwork from different angles encouraged participants to observe life experiences from different perspectives, as mentioned by this participant, *‘not only are you looking at the layers of the art, but it also plays into part of real life as well. Everyone here has different stories and different backgrounds. Just like the art piece, there’s the first perspectives and impressions of people, but you do not really know what their story is and what they have been through in their life. That’s kind of a life lesson when looking at slow art (SAP117, 28-year-old, male)*’. Moreover, hearing others’ interpretations of the artworks broadened their perspective, as one participant noted, ‘*I enjoyed the process of enjoying art as a group and hearing others’ perspectives. It shows me the deep and rich thought that each individual has, and I really appreciated that (SAP12, 30-year-old, female)*’.

#### 3.3.2 Theme 2: Insights to effective implementation (*n* = 134)

Participants shared insights into the aspects of the intervention that were most effective for them, as well as other factors that contributed to the overall effectiveness of the intervention.

Subtheme 2a: Valuable Components (*n* = 71). The mindfulness and self-compassion exercises, such as guided meditation, mindful breathing, and mindful movements, helped participants to relax and prioritize self-care. Some felt motivated to continue these practices in their daily lives after the session ended. This participant reflected on her experience, ‘*I appreciated that it allowed me to practice mindfulness – to slow down and fully immerse myself in the experience without distraction or interruption (SAP085, 21-year-old, female).* Additionally, the guided art appreciation activity encouraged observation and reflection, while the response art activity facilitated a deeper connection with the selected artwork. A participant wrote that ‘*naming the artwork allowed me to have a more personal connection with the artwork, and this simple act of creating something new is fulfilling (SAP070, 45-year-old, male)*’. Participants also valued the group discussion during the session, as it allowed them to share knowledge and interpretations with each other, as one participant explained, ‘*I realize when it comes to the group discussion, we have different perspectives … It’s good to be in a group so we can learn from one another (SAP199, 54-year-old, female)*’. Participants also felt that the components of the intervention were well integrated, as summarized below: ‘*It was packaged nicely, the narrative flow. Because you set the stage then you go into the activity of learning how to appreciate the art. And then after that, you go into the thought process of how you would interpret a piece. I thought it was nicely put together (SAP074, 36-year-old, female)*’.

Subtheme 2b: Execution Requisites (*n* = 80). Participants also discussed aspects of the program’s implementation that affected their experience. Particularly, they highlighted the crucial role of the facilitator in the intervention’s therapeutic effect, noting that facilitators provided guidance and created a safe space for vulnerability: ‘*I personally that feel the facilitator has been very important. Our current facilitator has done a very good job of setting the context and environment, despite having all these issues that we face. Being such a good facilitator has put us at ease and allow some of us to open up very easily. For this kind of course, the facilitator must be very well trained, and be just as good or better than our current facilitator. Once the environment is set and the context has been fixed with the parameters, it really makes people open up. And the whole thing just went very smoothly (SAP077, 57-year-old, male)*’. Participants specifically noted the facilitator’s calm demeanor, presentation style, and skill in summarizing group discussions as key factors in shaping their experience. They also valued the facilitator’s kind, patient, and compassionate approach, which created a sense of safety for sharing personal experiences. Additionally, participants appreciated the hospitality and professionalism of the liaison staff, which contributed to their positive experience of Slow Art Plus. Participants emphasized the importance of environmental factors in enhancing their overall experience. They preferred a private setting with minimal distractions and good acoustics to improve focus during the session. As this participant described, ‘*I find myself being calm at the beginning of the session and was more aware of my surroundings. But I also became increasingly stressed and annoyed by the endless motions around me and was unable to block it out like I could usually do … which disrupts this peace (SAP145, 32-year-old, female)*’. Moreover, participants highlighted the significance of logistical factors in enhancing comfort, such as having chairs with back support to alleviate discomfort during the session. Many participants found the gallery-issued stools uncomfortable for a 90-min session, and this sentiment was shared across different age groups, as one participant shared, ‘*I felt my back starting to hurt while sitting on the chair. Maybe changing the chair would be better (SAP007, 27-year-old, female)*’.

Subtheme 2c: Suggested Enhancements (*n* = 61). Some participants suggested that a single session might be too brief to observe lasting changes and proposed implementing multiple sessions with a variety of artworks, different mindfulness exercises, and longer group discussions. A participant expressed ‘*continuing over a longer term and having more than one session for each group would lead to further connectedness among participants (SAP168, 61-year-old, female)*’. On the artworks, another participant recommended ‘*having the process with another art piece of a different medium, allowing participants the opportunity to practice their slow art skillset (SAP084, 32-year-old, male)*’. A few participants also suggested providing take-home resources to continue practicing what they learned during the session. Additionally, some participants suggested offering light refreshments to enhance the overall experience. Finally, participants highly recommended Slow Art Plus to be a regular program in the gallery, appealing to the public through different segments (e.g., art education in schools, corporate activities, etc.) and expanding the curriculum to cater to the needs of diverse communities (e.g., caregivers, persons with health conditions or impairments, etc.). A participant suggested that ‘*this curriculum, self-care and mindfulness can be included in the primary school curriculum (SAP187, 61-year-old, female)’*.

## 4 Discussion

In line with the research objectives, a 90-min single session Slow Art Plus protocol was developed. The program’s effectiveness was evaluated using a wait-list randomized control trial design, and its acceptability was assessed through qualitative inquiry. This mixed-method approach provided valuable insights into the effects and implementation of the program. Although most of the mixed-model ANOVA results showed statistically insignificant changes in the primary and secondary outcomes, they underscored Slow Art Plus’s effectiveness in enhancing participants’ spiritual well-being, particularly in areas such as spiritual peace, life meaning, and faith. Furthermore, the repeated measures ANOVA analyses in the intervention group revealed significant reductions in perceived stress, along with an increase in mindfulness, active engagement with the world, and self-compassion. These findings suggest the possibility of lasting effects from the program and may also suggest that more time is needed for beneficial intrapersonal outcomes to emerge.

The qualitative data, gathered through acceptability focus groups and written feedback provided a nuanced understanding of the Slow Art Plus program’s impact and implementation processes. Participants reported experiencing improved peace and relaxation, which complemented the quantitative findings. They also demonstrated an increased sense of self-compassion and a commitment to prioritizing self-care, along with a broadening of perspectives. Regarding implementation components, participants found the self-compassion exercises, guided art appreciation, and group discussions to be particularly valuable. Additionally, key implementation factors such as the facilitator’s skill and presence, as well as the environment—including physical comfort and distractions—were noted to have an influence on the intervention and should be considered.

### 4.1 Interpreting results

The current study aimed to assess the overall effectiveness of the Slow Art Plus intervention without specifically examining the effects of each individual intervention component. Although the study did not isolate the contributions of each component, insights could be drawn from existing literature on the arts and humanities, mindfulness, and self-compassion.

There is limited research on interventions that combine art appreciation with mindfulness and self-compassion practices. However, the current findings contribute to the literature on the positive effects of museum and gallery-based interventions on mental, physical, emotional, and social well-being (57–60). The positive outcomes of this intervention may be explained by the RAISE (Reflection, Acquisition, Immersion, Socialization, and Expression) mechanisms in the conceptual model of how arts and humanities engagement contributes to human flourishing (61).

For instance, Slow Art Plus provides a platform for immersive engagement with artwork, enabling participants to fully absorb the art appreciation experience. This immersion may temporarily reduce awareness of surrounding circumstances, potentially broadening individuals’ experiences and contributing to their overall well-being (62). The opportunity for participants to express themselves creatively, such as through creating a playlist and response art, supported the expression of their feelings and thoughts, which has also been shown to positively impact well-being (63). Furthermore, both actively and passively engaging with the artwork could foster meaning-making among participants (47). This process of meaning-making through the arts could occur through affective, cognitive, and transpersonal symbolizing (64). In addition, the opportunities for discussion provided participants with a platform to be heard and feel accepted, affirming each person’s narrative and providing insight into the life experiences of others (65). This fostered socialization and enabled participants to form relationships and develop a shared identity (66), strengthening their sense of belonging which is known to contribute to well-being (67). Lastly, guided reflection in Slow Art Plus provided an opportunity for individuals to notice their internal- and external-focused thought (68), potentially leading to the development of new perspectives.

The mindfulness practices in Slow Art Plus provide a complementary approach to supporting well-being, with some components overlapping with arts and humanities engagement. The practice of mindfulness and mindfulness-based interventions has been empirically shown to promote self-compassion, emotional regulation, and improvements in various psychological indices (37, 69–71). The quantitative outcomes of improved mindfulness and reduced stress align with aspects of the Gallery’s Audience Engagement Framework, while the outcome of active engagements with the world (a resilience subscale) adds another dimension of psychological well-being, indicating the potential for individuals to actively engage in coping with stressful situations (72). Moreover, a high correlation was observed between the perceived stress scale and the subscale of perceived helplessness, which suggests that it may be a similar construct in this study. Perceived helplessness assesses an individual’s sense of control over their circumstances, emotions, and reactions (44). This correlation may indicate that the reduction in stress observed in the study was related to improvements in managing emotions and reactions, aligning with the potential benefits of mindfulness practices. The elements of mindfulness and self-compassion in Slow Art Plus helped participants non-judgmentally observe their present experiences with curiosity and acceptance, fostering a sense of calm and peace. This was reflected in both the quantitative and qualitative findings. The increased awareness of one’s emotional and mental states, along with the confidence to express thoughts and opinions, as well as the integration of life experiences through reminiscence, could contribute to improved mental health (73). Contrary to the hypothesis, the quantitative findings showed a decrease in the subscale of acting with awareness immediately after the intervention, which differed from the qualitative findings. One potential explanation could be participant fatigue at the end of the 90-min program, after intense concentration and focus. Lastly, the dyadic sharing and group conversations in the program not only strengthened relationships but deepened participant’s sense of common humanity, reassuring them that they are not alone in their struggles. This experience served as a gateway for them to nurture self-kindness. The findings suggested that the Slow Art Plus experience could be a helpful way to alleviate stress and strengthen one’s psychological resilience.

Finally, several important implementation factors were highlighted by research participants that should be considered when implementing similar interventions. Facilitators played a crucial role in creating a conducive environment for museum and mindfulness-based interventions (74). Drawing on Carl Rogers’ concept of unconditional positive regard, facilitators had the capacity to empower participants and influence psychological outcomes (75). By embodying qualities of presence, authenticity and empathy, Slow Art Plus facilitators fostered an environment conducive to self-awareness and personal growth (76). In addition, the significance of the physical and psychological space was echoed in prior research which suggested the gallery space’s role in encouraging creativity, exploration, and self-expression (77). Taken together, these discussions shed light on the effects of the Slow Art Plus’s effects and offer practical implementation strategies to support mental health promotion.

### 4.2 Limitations and implications for future projects

Firstly, in terms of research design, the use of a one-day waiting period for the control group was implemented to ensure that the questionnaires accurately represented the state of mind of the participants. However, the duration may be too short for meaningful comparisons which may have resulted in the current non-significant findings of the mixed model ANOVA. Additionally, the significant findings from the repeated measures ANOVA for the intervention group suggest that more time may be needed for the positive impact to become apparent. As a preliminary study, further research is necessary to confirm the positive impact of the program. Future investigations may consider extending the waiting period for the waitlist-control group and conducting a longer follow-up, including a one- to two-week waiting period and follow-up, to investigate maintenance effects. This could help evaluate the effects of slow looking and mindfulness activities core to the Slow Art Plus intervention protocol. In addition, incorporating multiple time points for future evaluations could provide insight into the long-term effects of the intervention. Although participants were made aware of their allocation outcomes after the baseline assessment, the blinding procedures could be improved as participants were aware of the research purpose and anticipated outcomes which may influence the research findings. To maintain blinding and reduce expectancy effects, future designs could include an active control group and an ethical narrative that maintains the integrity of the study while keeping the true objective undisclosed. Moreover, while the selection of the wait-list control groups for the focus group study was primarily a logistical consideration based on the availability of the activity rooms, it may raise concerns about bias in sampling. One possible solution could be to ensure that participants assigned to both groups are included for future studies, ensuring a more balanced distribution, and reducing the potential for bias.

Secondly, in the current analysis, the effect sizes observed for the intervention were small, indicating modest changes in measured outcomes. Additionally, the specific contributions of each component in the multi-modal intervention were not identified, suggesting that the combined effects of the intervention may not be adequately captured. This highlights the need for further investigation into the mechanisms underlying the intervention’s effects and the potential synergistic interactions between its components. Additionally, the high correlation between perceived stress and the subscale of perceived helplessness suggest that they may be measuring a similar construct, potentially indicating that the reduction in stress observed in the study is related to the management of emotions and reactions.

Thirdly, in terms of sampling, it is notable that the majority of the participants were female, of Chinese ethnicity, and highly educated, which may affect the generalizability of the findings. Future research could consider using a stratified random sampling method to ensure representation across a diverse range of genders, ethnicities, and socio-economic statuses. Moreover, as the preliminary findings shows promise in reducing stress, promoting self-care, and enhancing spiritual well-being, Slow Art Plus could be refined for specific populations which are recognized to have high stress levels such as formal and informal caregivers, healthcare professionals, and educators.

Finally, in terms of intervention design, the artwork used in the study could be expanded to apply the protocol with the gallery’s expansive range of Southeast Asian artworks. Comparative evaluation between art works and modalities could be explored further as research has suggested that specific elements within different types of artworks may be more suited for slow looking (78, 79). In addition, a multi-session Slow Art Plus could be further developed and tested using a longitudinal research design to investigate the intervention’s effectiveness across a longer period as suggested by some participants who expressed a desire for more sessions to explore various art and meditation techniques and to build stronger connections with other Slow Art Plus participants.

## 5 Conclusion

The empirical literature on the combined efficacy of museum and mindfulness-based interventions for mental health promotion are limited both in Singapore and internationally. Slow Art Plus is a unique, standardized, multimodal, single-session intervention that integrates slow-looking, mindfulness, and self-compassion practices, as well as reflective and creative expressions with Southeast Asian art. It shows promise in supporting mental health promotion for the general population and may be integrated into social prescribing programs for diverse backgrounds to improve spiritual well-being, mindfulness, self-compassion, and reduce stress. Slow Art Plus has the potential to introduce a new paradigm of mental health and self-care within the arts industry, offering vitality to individuals locally and around the world.

## Data availability statement

The original contributions presented in the study are included in the article/Supplementary material, further inquiries can be directed to the corresponding author. Requests to access the datasets should be directed to andyhyho@ntu.edu.sg.

## Ethics statement

The studies involving humans were approved by NTU Institutional Review Board (IRB). The studies were conducted in accordance with the local legislation and institutional requirements. Written informed consent for participation in this study was provided by the participants or the participants’ legal guardians/next of kin.

## Author contributions

AH, AT, and KP conceptualized and designed the study. AH and AT obtained funding. SM, GT-H, PC, JN, and AT was involved in the coordination and implementation of the research study. AH and KP delivered the intervention. JN, SM, PC, GT-H, and AH conducted the analysis. All authors contributed to data interpretation, as well as the writing and revision of the manuscript, contributed to the article, and approved the submitted version.
