# Supplementary material for: Slow art plus: developing and piloting a single session art gallery-based intervention for mental health promotion via a mixed method waitlist randomized control trial
Source: Front Public Health. 2024 May 13;12:1238564. doi: 10.3389/fpubh.2024.1238564 (PMC11128664; doi:10.3389/fpubh.2024.1238564)
Supplement: Supplementary file 1 [file Table_1.DOCX]

Supplementary Material

Slow Art Plus: Developing and Piloting a Single Session Art Gallery-based Intervention for Mental Health Promotion via a Mixed Method Waitlist Randomised Control Trial (RCT)

Ho, Andy Hau Yan^1,2,3*^, Ma, Stephanie Hilary Xinyi,^1*^, Ng, Jing Ting^1^, Choo, Ping Ying^1^, Tan-Ho, Geraldine^1*^, Pooh, Karen Chuan Ling^4^, Teng, Alicia^5^

^1^Action Research for Community Health Laboratory, Psychology Program, School of Social Sciences, Nanyang Technological University, Singapore.

^2^Lee Kong Chian School of Medicine, Nanyang Technological University, Singapore

^3^Palliative Care Centre for Excellence in Research and Education, Singapore

^4^ Department of Clinical, Educational and Health Psychology, University College London, UK

^5^Community & Access, National Gallery Singapore, Singapore.

*** Correspondence:**Corresponding Author
[andyhyho@ntu.edu.sg](mailto:andyhyho@ntu.edu.sg)

# Supplementary Figures and Tables

This document details the non-parametric tests conducted for variables that violate the normality assumption. a Mann-Whitney U test was conducted for between-group comparisons, while a Friedman test with follow-up Wilcoxon Signed-Rank Test was performed for within-group differences.

Table S 1 Between-group analysis using the Mann-Whitney U Test

| **Variables** | **T1** | | | | **T2** | | | | **T3** | | | |
| --- | --- | --- | --- | --- | --- | --- | --- | --- | --- | --- | --- | --- |
|  | **Immediate Intervention**  **Mean** | **Waitlist Control**  **Mean** | **U** | **Z** | **Immediate Intervention**  **Mean** | **Waitlist Control**  **Mean** | **U** | **Z** | **Immediate Intervention**  **Mean** | **Waitlist Control**  **Mean** | **U** | **Z** |
| Stress | 15.10 | 15.06 | 4781 | -0.05 | 14.64 | 14.13 | 4636.5 | -0.42 | 12.55 | 14.09 | 4060 | -1.87 |
| Quality of Life | 5.22 | 5.17 | 4692 | -0.29 | 5.35 | 5.15 | 4279.5 | -1.36 | 5.32 | 5.49 | 4430 | -0.98 |
| Self-Compassion | 3.27 | 3.67 | 4627 | -0.44 | 3.30 | 3.33 | 4563 | -0.60 | 3.38 | 3.47 | 4410.5 | -0.99 |
| Spiritual Well-Being | 32.22 | 32.15 | 4722 | -0.08 | 35.00 | 32.65 | 4087 | -1.80 | 33.94 | 35.31 | 4380 | -1.06 |

**p* <.05, ***p* <.001

Table S 2 Between-group analysis on subscales using the Mann-Whitney U Test

| **Variables** | | **T1** | | | | **T2** | | | | **T3** | | | |
| --- | --- | --- | --- | --- | --- | --- | --- | --- | --- | --- | --- | --- | --- |
|  |  | **Immediate Intervention**  **Mean** | **Waitlist Control**  **Mean** | **U** | **Z** | **Immediate Intervention**  **Mean** | **Waitlist Control**  **Mean** | **U** | **Z** | **Immediate Intervention**  **Mean** | **Waitlist Control**  **Mean** | **U** | **Z** |
| Stress | Perceived Helplessness | 9.58 | 9.41 | 4716.5 | -0.22 | 8.96 | 8.64 | 4670.0 | -0.33 | 7.72 | 8.85 | 3993.0 | -2.04* |
|  | Lack of Self-Efficacy | 5.52 | 5.65 | 4753.0 | -0.12 | 5.68 | 5.49 | 4569.0 | -0.59 | 4.83 | 5.24 | 4325.5 | -1.21 |
| Mindfulness | Observing | 14.91 | 14.63 | 4537.0 | -0.67 | 14.99 | 14.69 | 4613.0 | -0.48 | 15.81 | 15.79 | 4723.0 | -0.20 |
|  | Describing | 13.56 | 13.63 | 4659.0 | -0.36 | 14.05 | 13.37 | 4215.5 | -1.49 | 14.30 | 14.23 | 4756.5 | -0.12 |
|  | Acting with Awareness | 13.49 | 13.58 | 4533.5 | -0.68 | 12.86 | 13.32 | 4376.0 | -1.08 | 13.51 | 13.51 | 4755.0 | -0.12 |
|  | Nonjudging to inner experience | 12.52 | 12.55 | 4692.5 | -0.28 | 12.14 | 12.30 | 4576.0 | -0.57 | 12.99 | 12.99 | 4792.5 | -0.02 |
|  | Nonreacting to inner experience | 12.87 | 13.11 | 4625.5 | -0.45 | 13.41 | 12.89 | 4260.5 | -1.38 | 13.58 | 13.44 | 4768.5 | -0.09 |
| Resilience | Integrated performance under stress | 3.02 | 2.95 | 4379.0 | -1.10 | 2.97 | 2.92 | 4458.5 | -0.91 | 3.00 | 3.04 | 4604.0 | -0.53 |
|  | Active Engagement with the world | 3.15 | 3.01 | 4074.5 | -1.84 | 3.17 | 2.99 | 3860.5 | -2.39 | 3.26 | 3.15 | 4234.5 | -1.44 |
|  | Repertoire of cognitive, social and personal problem-solving strategies | 2.96 | 2.97 | 4709.5 | -0.24 | 2.97 | 2.95 | 4753.5 | -0.12 | 2.98 | 3.03 | 4556.5 | -0.63 |
| Self-Compassion | Self-kindness, Self-judgement | 3.33 | 3.41 | 4527.0 | -0.70 | 3.33 | 3.37 | 4561.0 | -0.61 | 3.43 | 3.51 | 4361.5 | -1.12 |
|  | Common humanity, Isolation | 3.24 | 3.31 | 4618.5 | -0.47 | 3.29 | 3.30 | 4584.5 | -0.55 | 3.35 | 3.42 | 4534.5 | -0.68 |
|  | Mindfulness, Over-identification | 3.24 | 3.38 | 4484.0 | -0.81 | 3.30 | 3.31 | 4754.0 | -0.12 | 3.36 | 3.46 | 4510.5 | -0.74 |
| Spiritual Wellbeing | Meaning | 11.88 | 11.80 | 4644.5 | -0.40 | 12.44 | 11.88 | 4317.5 | -1.23 | 12.38 | 12.58 | 4638.0 | -0.42 |
|  | Peace | 10.56 | 10.47 | 4707.0 | -0.24 | 11.72 | 10.59 | 3795.5 | -2.55 | 11.09 | 11.98 | 4060.0 | -1.88 |
|  | Faith | 9.79 | 9.89 | 4742.5 | -0.15 | 10.84 | 10.18 | 4427.0 | -0.95 | 10.47 | 10.74 | 4568.0 | -0.59 |

**p* <.05, ***p* <.001

Table S 3 Within-group analysis using the Friedman test with follow-up Wilcoxon Signed Rank Test

| **Variable** | | **Friedman** | **T1 vs T2** | | | **T1 vs T3** | | |
| --- | --- | --- | --- | --- | --- | --- | --- | --- |
|  |  | **χ^2^** | **W^-^** | **W^+^** | **Z** | **W^-^** | **W^+^** | **Z** |
| Stress | Immediate Intervention | 19.57** | 2082.0 | 1659.0 | -0.92 | 3175.0 | 1103.0 | -4.04** |
|  | Waitlist Control | 8.94* | 2683.5 | 1594.5 | -2.13* | 2174.0 | 1147.0 | -2.42* |
| Quality of Life | Immediate Intervention | 2.83 | 352.5 | 508.5 | -1.06 | 547.5 | 677.5 | -0.70 |
|  | Waitlist Control | 20.88** | 425.5 | 394.5 | -0.22 | 287.0 | 841.0 | -3.08* |
| Self-Compassion | Immediate Intervention | 6.77* | 1704.5 | 1950.5 | -0.54 | 1471.0 | 2715.0 | -2.47* |
|  | Waitlist Control | 7.56* | 2359.0 | 1736.0 | -1.26 | 1478.0 | 2708.0 | -2.44* |
| Spiritual Well-Being | Immediate Intervention | 14.52** | 752.5 | 2650.5 | -4.40** | 1234.5 | 2681.5 | -3.02* |
|  | Waitlist Control | 34.97** | 1500.0 | 2416.0 | -1.92 | 695.0 | 3221.0 | -5.27** |

**p* <.05, ***p* <.001

Table S 4 Within-group analysis on subscales using the Friedman test with follow-up Wilcoxon Signed Rank Test

|  | **Variable** | | | **Friedman** | **T1 vs T2** | | | **T1 vs T3** | | |
| --- | --- | --- | --- | --- | --- | --- | --- | --- | --- | --- |
|  |  |  |  | **χ^2^** | **W^-^** | **W^+^** | **Z** | **W^-^** | **W^+^** | **Z** |
| Stress | | Perceived Helplessness | Immediate Intervention | 18.00** | 2116.5 | 1538.5 | -1.27 | 2796.0 | 774.0 | -4.52** |
|  |  |  | Waitlist Control | 6.11* | 2102.0 | 1219.0 | -2.09* | 1832.0 | 1094.0 | -1.92 |
|  |  | Lack of Self-Efficacy | Immediate Intervention | 10.65* | 1331.5 | 1518.5 | -0.50 | 1788.0 | 987.0 | -2.18* |
|  |  |  | Waitlist Control | 4.81 | 1653.0 | 1428.0 | -0.57 | 1871.5 | 1131.5 | -1.91 |
| Mindfulness | | Observing | Immediate Intervention | 21.62** | 1623.5 | 1697.5 | -0.18 | 606.0 | 1950.0 | -3.89** |
|  |  |  | Waitlist Control | 29.90** | 1154.5 | 1260.5 | -0.32 | 441.0 | 2260.0 | -5.07** |
|  |  | Describing | Immediate Intervention | 5.39 | 955.0 | 1673.0 | -2.04* | 640.0 | 1706.0 | -3.30** |
|  |  |  | Waitlist Control | 24.84** | 1405.0 | 941.0 | -1.44 | 669.0 | 1677.0 | -3.12* |
|  |  | Acting with Awareness | Immediate Intervention | 11.28* | 2010.0 | 993.0 | -2.63* | 1254.5 | 1373.5 | -0.34 |
|  |  |  | Waitlist Control | 0.24 | 1386.0 | 960.0 | -1.32 | 1823.5 | 1746.5 | -0.17 |
|  |  | Nonjudging to inner experience | Immediate Intervention | 7.02* | 1673.0 | 1253.0 | -1.10 | 1120.0 | 1730.0 | -1.62 |
|  |  |  | Waitlist Control | 3.92 | 1592.0 | 1183.0 | -1.12 | 1139.5 | 1863.5 | -1.86 |
|  |  | Nonreacting to inner experience | Immediate Intervention | 3.92 | 1170.5 | 2232.5 | -2.51* | 1035.0 | 2451.0 | -3.27* |
|  |  |  | Waitlist Control | 4.81 | 1419.5 | 1136.5 | -0.82 | 702.5 | 1250.5 | -1.94 |

**p* <.05, ***p* <.001

Table S 4 Within-group analysis on subscales using the Friedman test with follow-up Wilcoxon Signed Rank Test (cont’d)

|  | **Variable** | | | **Friedman** | **T1 vs T2** | | | **T1 vs T3** | | |
| --- | --- | --- | --- | --- | --- | --- | --- | --- | --- | --- |
|  |  |  |  | **χ^2^** | **W^-^** | **W^+^** | **Z** | **W^-^** | **W^+^** | **Z** |
| Resilience | | Integrated performance under stress | Immediate Intervention | 3.16 | 723.0 | 502.0 | -1.19 | 799.5 | 685.5 | -0.52 |
|  |  |  | Waitlist Control | 7.77* | 687.0 | 588.0 | -0.51 | 451.5 | 773.5 | -1.70 |
|  |  | Active engagement with the world | Immediate Intervention | 12.95* | 1267.0 | 1508.0 | -0.67 | 926.5 | 2154.5 | -3.12* |
|  |  |  | Waitlist Control | 15.77** | 1320.0 | 1165.0 | -0.46 | 628.0 | 1787.0 | -3.51** |
|  |  | Repertoire of cognitive, social and personal problem-solving strategies | Immediate Intervention | 0.84 | 1149.5 | 1265.5 | -0.36 | 922.5 | 1093.5 | -0.60 |
|  |  |  | Waitlist Control | 7.54* | 1214.0 | 1064.0 | -0.48 | 802.0 | 1343.0 | -1.82 |
| Self-Compassion | | Self-kindness, Self-judgement | Immediate Intervention | 8.08* | 1411.0 | 1290.0 | -0.34 | 1131.0 | 1872.0 | -1.91 |
|  |  |  | Waitlist Control | 3.56 | 1604.0 | 1322.0 | -0.74 | 1344.0 | 1896.0 | -1.34 |
|  |  | Common humanity, Isolation | Immediate Intervention | 5.91 | 1391.0 | 1690.0 | -0.76 | 949.0 | 1536.0 | -1.73 |
|  |  |  | Waitlist Control | 3.83 | 1555.5 | 1447.5 | -0.28 | 912.0 | 1644.0 | -2.14* |
|  |  | Mindfulness, Over-identification | Immediate Intervention | 3.18 | 1343.5 | 1659.5 | -0.82 | 1347.5 | 2222.5 | -1.97* |
|  |  |  | Waitlist Control | 6.47* | 1670.0 | 1256.0 | -1.09 | 1103.0 | 1598.0 | -1.38 |
| Spiritual Wellbeing | | Meaning | Immediate Intervention | 1.81 | 784.0 | 1427.0 | -2.09* | 817.5 | 1528.5 | -2.22* |
|  |  |  | Waitlist Control | 15.80** | 948.0 | 1263.0 | -1.02 | 443.0 | 1702.0 | -4.17** |
|  |  | Peace | Immediate Intervention | 10.05* | 878.5 | 2281.5 | -3.45** | 1105.5 | 1820.5 | -1.87 |
|  |  |  | Waitlist Control | 28.02** | 1134.0 | 1351.0 | -0.65 | 582.5 | 2343.5 | -4.59** |
|  |  | Faith | Immediate Intervention | 27.71** | 363.5 | 1847.5 | -4.81** | 481.5 | 1348.5 | -3.23* |
|  |  |  | Waitlist Control | 16.32** | 1033.5 | 1451.5 | -1.25 | 671.5 | 1956.5 | -3.66** |

**p* <.05, ***p* <.001
